# Modulation of SETDB1 activity by APQ ameliorates heterochromatin condensation, motor function, and neuropathology in a Huntington’s disease mouse model

**DOI:** 10.1080/14756366.2021.1900160

**Published:** 2021-03-26

**Authors:** Yu Jin Hwang, Seung Jae Hyeon, Younghee Kim, Sungsu Lim, Min Young Lee, Jieun Kim, Ashwini M. Londhe, Lizaveta Gotina, Yunha Kim, Ae Nim Pae, Yong Seo Cho, Jihye Seong, Hyemyung Seo, Yun Kyung Kim, Hyunah Choo, Hoon Ryu, Sun-Joon Min

**Affiliations:** a Brain Science Institute, Korea Institute of Science and Technology (KIST), Seoul, Republic of Korea; b Convergence Research Center for Diagnosis, Treatment and Care System of Dementia, KIST, Seoul, Republic of Korea; c Institute for Systems Biology, Seattle, WA, USA; d Division of Bio-Medical Science & Technology, KIST School, Korea University of Science and Technology, Seoul, Republic of Korea; e Department of Molecular & Life Sciences, Center for Bionano Intelligence Education and Research, Hanyang University, Ansan, Republic of Korea; f Department of Neurology and Boston University Alzheimer’s Disease Center, Boston University School of Medicine, Boston, MA, USA; g Department of Chemical & Molecular Engineering/Applied Chemistry, Center for Bionano Intelligence Education and Research, Hanyang University, Ansan, Republic of Korea

**Keywords:** Histone H3K9me3-specific transferase, SETDB1, Huntington’s disease, medium spiny neuron, motor function

## Abstract

The present study describes evaluation of epigenetic regulation by a small molecule as the therapeutic potential for treatment of Huntington’s disease (HD). We identified 5-allyloxy-2-(pyrrolidin-1-yl)quinoline (APQ) as a novel SETDB1/ESET inhibitor using a combined *in silico* and *in vitro* cell based screening system. APQ reduced SETDB1 activity and H3K9me3 levels in a HD cell line model. In particular, not only APQ reduced H3K9me3 levels in the striatum but it also improved motor function and neuropathological symptoms such as neuronal size and activity in HD transgenic (YAC128) mice with minimal toxicity. Using H3K9me3-ChIP and genome-wide sequencing, we also confirmed that APQ modulates H3K9me3-landscaped epigenomes in YAC128 mice. These data provide that APQ, a novel small molecule SETDB1 inhibitor, coordinates H3K9me-dependent heterochromatin remodelling and can be an epigenetic drug for treating HD, leading with hope in clinical trials of HD.

## Graphical Abstract

**Figure:**
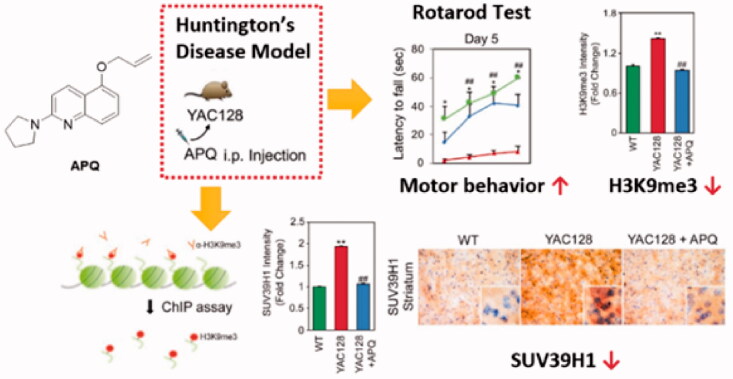

## Introduction

Huntington’s disease (HD) is a genetic neurodegenerative disease characterised by progressive neuronal loss, motor abnormality, cognitive impairment, and neuropsychiatric disorders^1^. The main cause of HD is an expanded copy of the CAG trinucleotides repeat at the 5′ terminal region of the Huntingtin (HTT) gene, which is translated to a polyglutamine expansion in the HTT protein^2^. Although the function of HTT in humans is not clearly understood, it is usually expressed in the body and brain and its mutation produces excitotoxicity, mitochondrial dysfunctions, axonal transport deficit, altered proteasome activity, and gene dysregulation^3^^,^^4^. Recent research has shown that the multiple cellular and molecular alterations are involved in the pathogenesis of HD, but a prevailing pathway from mutated HTT to neuronal dysfunction still remains to be established. There are at present no clinically validated therapeutic agents that slow or halt disease progression in HD. However, epigenetic modulators such as histone deacetylases (HDACs) inhibitors have been found to show significant neuroprotective effects in HD cellular and mouse models^5–13^, which suggests that histone modifications may be potential therapeutic targets for HD^14^.

Histone methyl transferases (HMTs) are the enzymes that catalyse methylation of lysine or arginine residues of the histone protein tail^15–19^. The HMTs have received much attention from the perspective of drug discovery in recent years because their enzymatic actions are responsible for various physiological functions through controlling gene transcription^11–13^^,^^15^^,^^16^^,^^20–23^. It has become evident that abnormal expression or activity of the HMTs are associated with pathogenesis of cancer, inflammation, and neurodegenerative diseases. SET domain bifurcated 1 (SETDB1) belongs to the SET domain-containing protein methyltransferase family, which is involved in tri-methylation of histone H3 on lysine 9 position (H3K9)^17^^,^^18^^,^^24–26^. SETDB1 interacts with various enzymes and transcription factors to mediate transcriptional repression or gene silencing via its HMT activity. SETDB1 also plays an important role in the early development of mouse embryos.

**Figure 1.**
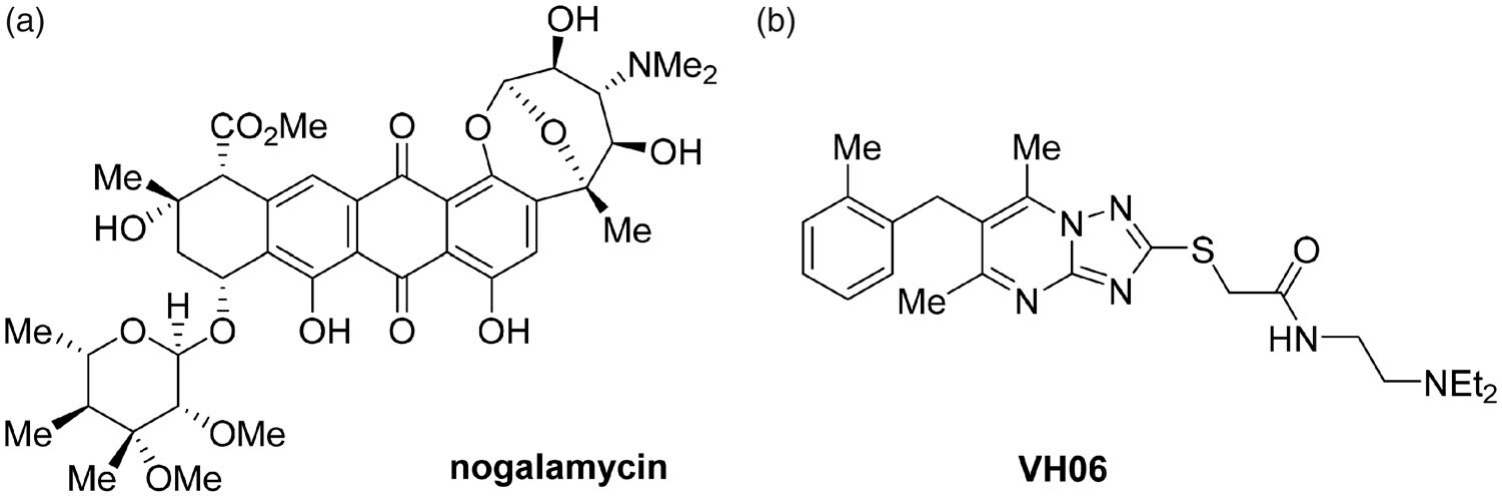
Structures of recently reported SETDB1 inhibitors, nogalamycin and VH06.

In our previous studies, we have reported that both SEDB1 expression and H3K9m3 levels were elevated in R6/2 transgenic mice and in HD patients^24^. Pharmacological treatment with transcriptional suppressors such as mithramycin and cystamine down-regulates SETDB1 gene expression and reduced H3K9 trimethylation, which improved behaviour and neuropathological phenotype in HD transgenic mice^24^^,^^27^. Further studies confirmed that nogalamycin (Figure 1(a)), another DNA binding antibiotic, showed the improved neuroprotective effects in HD transgenic mice by suppressing elevated SETDB1 and H3K9me3 level^24^^,^^28^. On the other hand, *in silico* approach also identified a small molecule VH06 (Figure 1(b)) from virtual screening, which reduced H3K9me3 without affecting SETDB1 protein expression and mRNA levels. Although both compounds restored neuropathological abnormalities *in vitro* or *in vivo* by inhibition of SETDB1 activity, they could not be further developed as lead compounds because it is difficult to modify nogalamycin due to its complex structure and *in vivo* evaluation of VH06 is not confirmed yet.

Accordingly, our goal is to screen a focussed chemical library to identify a new amenable scaffold of SETDB1 inhibitors and investigate the effects of this lead compound on SETDB1 regulation in cells and heterochromatin condensation in transgenic mice models of HD. In addition, we will examine inhibitory effects of our compound on both SETDB1 enzymatic activity and promoter activity. Thus, this study will highlight epigenetic modification by a small molecule as the therapeutic potential for treatment of HD.

## Materials and methods

### General

All reactions were conducted under oven-dried glassware under an atmosphere of nitrogen. All commercially available reagents were purchased and used without further purification. Solvents and gases were dried according to standard procedures. Organic solvents were evaporated with reduced pressure using a rotary evaporator. Reactions were followed by analytical thin layer chromatography (TLC) analysis using glass plates precoated with silica gel (0.25 mm). TLC plates were visualised by exposure to UV light (UV), and then were visualised with a KMnO_4_ or *p*-anisaldehyde stain followed by brief heating on hot plate. Flash column chromatography was performed using silica gel 60 (230–400 mesh, Merck, Kenilworth, NJ) with the indicated solvents. ^1^H and ^13^C spectra were recorded on Bruker 300 or Bruker 400 NMR spectrometers (Billerica, MA). ^1^H NMR spectra are represented as follows: chemical shift, multiplicity (s = singlet, d = doublet, t = triplet, q = quartet, m = multiplet), integration, and coupling constant (*J*) in Hertz (Hz). ^1^H NMR chemical shifts are reported relative to CDCl_3_ (7.26 ppm). ^13^C NMR was recorded relative to the central line of CDCl_3_ (77.0 ppm). GC/MS analyses were performed on Agilent 6890N Network GC system (Santa Clara, CA). The analysis of HPLC purity was performed on a Waters Alliance System with UV detector set to at 254 and 280 nm (Milford, MA). Samples were injected (10 µl) onto a Waters Sunfire 4.6 × 150 mm, 5.0 µM, C18 column maintained at 25.8 °C. A linear gradient from 30% to 100% B (MeCN) in 20 min was followed by pumping 100% B for another 10 min with A being H_2_O + 0.1 M NH_4_OAc (or NH_4_HCO_2_). The flow rate was 1.0 ml/min.

### Synthesis of 5-allyloxy-2-(pyrrolidin-1-yl)quinolone (APQ)

#### 5-Allyloxyquinoline (2)

After adding sodium hydride (60% dispersion in mineral oil, 90 mg, 2.25 mmol) to a round-bottomed flask set to 0 °C using ice and water, DMF (17.4 ml) was slowly added. Then, 5-hydroxylquinoline **1** (252 mg, 1.74 mmol) dissolved in DMF (4.0 ml) was slowly added to the round-bottomed flask. The temperature of the reaction mixture was raised to room temperature for 10 min. Ally bromide (0.165 ml, 1.91 mmol) was slowly added to the resulting yellow reaction mixture. After confirming the termination of reaction by TLC (ethyl acetate/hexane = 1:4, *R*_f_=0.5), H_2_O was added to the reaction mixture. The reaction mixture was separated into an ethyl acetate layer and an H_2_O layer using a separatory funnel. After drying the organic layer with anhydrous MgSO_4_, the solvent was removed by vacuum distillation. The mixture was purified by column chromatography on silica gel (ethyl acetate/hexane = 1:4) to obtain the target compound (yellow oil, 296 mg, 92%). ^1^H NMR (CDCl_3_, 300 MHz) *δ* 8.90 (dd, *J*_1_=4.2 Hz, *J*_2_=1.7 Hz, 1H), 8.62 (dt, *J*_1_=8.5 Hz, *J*_2_=0.8 Hz, 1H), 7.70 (d, *J* = 8.5 Hz, 1H), 7.59 (t, *J* = 8.1 Hz, 1H), 7.37 (dd, *J*_1_=8.5 Hz, *J*_2_=4.2 Hz, 1H), 6.85 (d, *J* = 7.7 Hz, 1H), 6.22–6.09 (m, 1H), 5.51 (dt, *J*_1_=17.3 Hz, *J*_2_=1.5 Hz, 1H), 5.35 (dq, *J*_1_=10.5 Hz, *J*_2_=1.3 Hz, 1H), 4.71 (dt, *J*_1_=5.1 Hz, *J*_2_=1.4 Hz, 2H). ^13^C NMR (CDCl_3_, 100 MHz) *δ* 153.9, 150.5, 149.0, 132.8, 130.7, 129.2, 121.6, 120.8, 120.1, 117.6, 105.3, 69.0. GC/MS: *m/z* (EI) 185 (M^+^).

#### 5-Allyloxy-2-chloroquinoline (3)

After dissolving 5-(allyloxy)quinoline **2** (296 mg, 1.60 mmol) in dichloromethane (8.0 ml) and then adding *m*CPBA (406 mg, 1.34 mmol) at 0 °C, the mixture was stirred at room temperature for 2 h. After confirming the formation of *N*-oxide by TLC (ethyl acetate/hexane = 1:4, *R*_f_=0.1), H_2_O was slowly added. The reaction mixture was separated into a dichloromethane layer and an H_2_O layer using a separatory funnel. After drying the organic layer with anhydrous MgSO_4_, the solvent was removed by vacuum distillation. After dissolving the dried *N*-oxide mixture in dichloromethane (8.0 ml), POCl_3_ (0.22 ml, 2.36 mmol) was slowly added. The reaction mixture was stirred at 50 °C for 7 h. After confirming the termination of reaction by TLC (ethyl acetate/hexane = 1:4, *R*_f_=0.7), H_2_O was slowly added. The reaction mixture was separated into a dichloromethane layer and an H_2_O layer using a separatory funnel. After drying the organic layer with anhydrous MgSO_4_, the solvent was removed by vacuum distillation. The crude mixture was purified by column chromatography on silica gel (ethyl acetate/hexane = 1:4) to obtain the target compound **3** (colourless oil, 138 mg, 39%). ^1^H NMR (CDCl_3_, 300 MHz) *δ* 8.56 (d, *J* = 8.7 Hz, 1H), 7.61 (d, *J* = 5.6 Hz, 1H), 7.61 (d, *J* = 3.1 Hz, 1H), 7.36 (d, *J* = 8.7 Hz, 1H), 6.88 (dd, *J*_1_=5.6 Hz, *J*_2_=3.1 Hz, 1H), 6.21–6.09 (m, 1H), 5.50 (dd, *J*_1_=17.3 Hz, *J*_2_=1.5 Hz, 1H), 5.37 (dd, *J*_1_=10.5 Hz, *J*_2_=1.3 Hz, 1H), 4.72 (dt, *J*_1_=5.2 Hz, *J*_2_=1.4 Hz, 2H). ^13^C NMR (CDCl_3_, 100 MHz) *δ* 154.1, 151.2, 148.8, 134.0, 132.6, 130.4, 122.0, 121.2, 119.4, 118.1, 106.0, 69.3. GC/MS: *m/z* (EI) 219 (M^+^).

#### 5-Allyloxy-2-(pyrrolidin-1-yl)quinoline (APQ, 4)

After adding 5-(allyloxy)-2-chloroquinoline **3** (33 mg, 0.15 mmol) to a vial, pyrrolidine (190 µl, 2.28 mmol) was slowly added. The reaction mixture was stirred at 140 °C for 12 h. After confirming the termination of reaction by TLC, H_2_O was slowly added. The reaction mixture was separated into an ethyl acetate layer and an H_2_O layer using a separatory funnel. After drying the organic layer with anhydrous MgSO_4_, the solvent was removed by vacuum distillation. The mixture was purified by column chromatography on silica gel (ethyl acetate/hexane = 1:4) to obtain the target compound **4** (white solid, 29 mg, 76%). ^1^H NMR (CDCl_3_, 400 MHz) *δ* 8.30 (d, *J* = 9.2 Hz, 1H), 7.39 (t, *J* = 8.1 Hz, 1H), 7.31 (d, *J* = 8.4 Hz, 1H), 6.67 (d, *J* = 9.2 Hz, 1H), 6.54 (d, *J* = 7.6 Hz, 1H), 6.20–6.10 (m, 1H), 5.49 (dd, *J*_1_=17.3 Hz, *J*_2_=1.6 Hz, 1H), 5.32 (dd, *J*_1_=10.5 Hz, *J*_2_=1.4 Hz, 1H), 4.66 (dd, *J*_1_=5.1 Hz, *J*_2_=1.4 Hz, 2H), 3.63–3.60 (m, 4H), 2.03–2.00 (m, 4H). ^13^C NMR (CDCl_3_, 100 MHz) *δ* 156.1, 154.5, 149.6, 133.5, 131.6, 129.2, 119.0, 117.3, 114.3, 108.9, 101.5, 68.9, 46.8, 25.6. GC/MS: *m/z* (EI) 254 (M^+^). HPLC purity: 98.74%.

### Homology modelling

Homology model of the SET domain of SETDB1 (amino acids 792–1291) was taken from our previous study^29^. Docking study was performed using the Gold suit-5.2^30^. Docking has performed using the gold wizard with CHEMPLP score as a scoring function. Images were prepared using Discovery studio-2018 software^31^.

### Histone extraction and dot blot analysis

Cells were homogenised with Dounce homogeniser in 500 ml of phosphate-buffered saline containing 0.4 mM sodium butyrate, 5% Triton X-100, 3 mM DTT, 1 mM sodium orthovanadate, 5 mM sodium fluoride, 3 mM PMSF, 3 mM DTT, 0.5 mg/ml leupeptin, and 10 mg/ml aprotinin as previously described^32–34^. The nuclear pellets were collected and washed twice with the above-described 5% Triton buffer. Histones were extracted by solubilising in 200 ml of 0.2 M HCl on a shaker for 2 h. After neutralising the pH of the acid-extracted solution containing the histone pool with ammonium acetate, the protein content was quantified. Each histone extract (an amount of 10 mg/20 ml) was placed onto each well of the dot blot apparatus pre-assembled with a nitrocellulose membrane and vacuumed for 30 min. After releasing the vacuum, the nitrocellulose membrane was removed and washed twice with TBS-T for 5 min. Then, the nitrocellulose membrane was blocked with 5% milk/TBS-T for 30 min and subsequently incubated with primary antibody in 5% milk/TBS-T for 24 h at cold room. The nitrocellulose membrane was washed twice with TBS-T for 5 min and incubated with secondary antibody in 1% milk (15 ml) for one hour at room temperature. After washing the membrane twice with TBS-T for 5 min, the immune reactivity was developed with Chemiluminescent Kit.

### Tet-inducible SETDB1/ESET cell line

The T-RExTM System (Invitrogen, Carlsbad, CA)-based inducible SETDB1/ESET cell line was established as previously described^21^^,^^28^. This system utilised two vectors, the pcDNA6/TR vector, a regulatory plasmid that expresses the tetracycline repressor (TetR), and pcDNA5/TO that contains a CMV promoter driving the expression of the gene of interest under the control of Tet-operator sequences. Myc-ESET was subcloned into the pcDNA5/TO vector from pcDNA-Myc-ESET construct, in which full length of ESET is cloned to a CMV-driven vector (Clontech, Palo Alto, CA). pcDNA5/TO-Myc-ESET was linearised and transfected into Q7 striatal cell clone over expressing pcDNA6/TR. The SETDB1/ESET cell clones were selected by hygromycin. For the induction of SETDB1/ESET, 4 µg/ml of doxycycline (Doxy) was treated into culture medium.

### Cell culture and SETDB1/ESET promoter assay

ST*Hdh*^Q7/Q7^ (wild type) and ST*Hdh*^Q111/Q111^ (HD knock-in striatal cell line expresses mutant huntingtin at endogenous level) were generously provided from Dr. Marcy MacDonald (Harvard Medical School, Boston, MA)^35^. The SETDB1/ESET promoter assay was performed as previously described^24^^,^^28^. Briefly, 2.5 × 10^5^ cells were plated onto 48-well cell culture plates 24 h prior to the transfection of 200 ng of ETDB1/ESET promoter construct. DMRIE-C reagent (Invitrogen, Carlsbad, CA) was used as a transfection reagent and transfections were performed according to the manufacturer’s protocol. Nogalamycin and distamycin were purchased from Sigma-Aldrich (St. Louis, MO).

### Histone H3K9 fluorescence resonance energy transfer biosensor and live cell imaging

Fluorescence resonance energy transfer (FRET)-based histone H3K9 methylation biosensor was previously described^36^. In brief, H3K9 biosensor is composed of a methyl-lysine binding domain of HP1 protein and a H3 tail peptide containing lysine9 between ECFP and YPet, and the NLS sequences were added in the C-terminal for the nucleus localisation of the biosensor. For the image acquisition, Q7 or Q111 cells were cultured on cover-glass-bottom dishes and the H3K9 biosensor was transfected into the cells using Lipofectamine 2000 (Invitrogen, Carlsbad, CA) according to the manufacturer’s protocols. Images were collected by a Nikon Ti-E inverted microscope with 420DF20 excitation filter, 450DRLP dichroic mirror, and two emission filters (475DF40 for ECFP and 535DF25 for YPet) controlled by a filter changer. Image analysis was performed using NIS element-AR software. Nucleus regions were selected as the regions of interests and the emission ratio of ECFP and YPet was calculated after background subtraction. The colour of each pixel was determined by the ECFP/YPet ratio value.

### Chemiluminescence SETDB1 enzyme assay

SETDB1 enzyme activity was analysed using commercially available SETBD1 chemiluminescent assay kit (BPS Bioscience, San Diego, CA, #51056) according to the manufacturer’s instructions. All analyses were performed in duplicates and all reagents used are supplied in the kit. *S*-adenosyl-l-homocysteine (SAH) as a product of *S*-adenosyl-l-methionine (SAM)-dependent transmethylation reaction was used as a non-selective inhibitor of SETDB1. Briefly, SAH and test compound APQ were diluted serially in DMSO and added at a final concentration of 1–100 µM in threefold increment. Total 50 µL mixture of histone methylation transfer buffer (50 mM Tris–HCl, pH 9.0, 1 mM phenylmethylsulfonyl fluoride, and 0.5 mM dithiothreitol) containing 20 µM SAM, each concentration of inhibitors and 1.25 ng/µL SETDB1 enzyme was added to each well of a 96-well plate. The wells were pre-coated with histone H3 peptide substrate. Reactions were allowed to proceed for 1 h 30 min at room temperature. Methylated K9 residue of H3 peptide was detected with anti-methylated H3K9 primary antibody and HRP-conjugated secondary antibody. Chemiluminescence resulting from the reaction between the HRP-conjugated secondary antibody and the added HRP substrate was measured using a SpectraMax M2 plate reader (Molecular Devices, Sunnyvale, CA).

### Cell viability assay

Cell viability was quantified using MTT assay (Sigma, St. Louis, MO, M2128). In brief, cells were cultured in each well of 96-well plates with varying concentrations of compounds for 24 h. Following incubation of cells in each well with MTT (0.5 mg/ml) for 4 h, the culture supernatant was discarded. Cells were washed with PBS three times, and the insoluble formazan product dissolved in DMSO. The optical density (OD) of each culture well was measured using as ELISA reader at 570 nm. The OD570 in control cells was taken as 100% viability.

### Animals and drug administration

YAC128 mice were bred with females from their background strain (B6CBAFI/J), and offspring were genotyped using PCR as previously described^37–41^. All mice were weighed at 3 weeks of age and equally distributed according to weight and percentage within each cohort. APQ (5 mg/kg/d) was administered by i.p. injection five times a week at the same time of day for all groups at 7 months of age. The biochemical and behavioural/neuropathological symptoms of mice were determined after 4 weeks of drug administration. These procedures were performed in accordance with Guide for the Care and Use of Laboratory Animals guidelines. The animal protocol was approved by IACUC (Institutional Animal Care and Use Committee) in Hanyang University, Ansan, Republic of Korea.

### Confocal microscopy and immunohistochemistry

Immunofluorescence staining and confocal microscopy was used to determine the trimethylated-histone H3 (Lys9) (H3K9me3) (Millipore, Billerica, MA) (1:1000). The procedures were performed as previously described^32–34^. The specimens were incubated for 1 h with Alexa Fluor 594 goat anti-rabbit antibody (abcam, Cambridge, UK; 1:400) after incubation of the primary antibody. Images were analysed using an A1 Nikon confocal laser scanning microscope (Nikon, Tokyo, Japan).

### Chromatin immunoprecipitation (ChIP)

ChIP for H3K9me3 binding to DNA was performed using a CHIP assay kit (Santa Cruz, Dallas, TX) as described previously^21^^,^^28^. Striatal tissues from WT, vehicle treated YAC128, and APQ-treated YAC128 mice were crossed-linked with 1% formaldehyde for 20 min at room temperature. Lysates were sonicated for 30 s six times using a Bioruptor (Diagenode Inc., Denville, NJ). After centrifugation, the supernatant was diluted in CHIP dilution buffer and incubated overnight at 4 °C with anti-H3K9me3 antibody. Immune complexes were recovered by the addition of 60 µl of salmon sperm DNA/protein A agarose-50% slurry and incubation at 4 °C for 2 h with rotation. Beads were pelleted and washed with low and high salt buffer followed by LiCl buffer (x3). Immune complexes were eluted by incubation with 500 µl of fresh elution buffer (1% SDS, 0.1 M NaHCO_3_) and 20 µl of 5 M NaCl. DNA was cleaned by the addition of 500 mM EDTA, 1 M Tris, and proteinase K at 66 °C for 4 h. The DNA solution was extracted with a phenol/chloroform/isoamyl alcohol mixture to remove protein contaminants, followed by precipitation with 100% ethanol. After precipitation, the pellet was washed with 70% ethanol and dissolved in 20 µl DW.

### Identification of differentially modified regions (DMRs)

The Model-based Analysis of ChIP-Seq algorithm (MACS; v. 1.4.2) was used to identify regions of H3K9me3 enrichment with the default parameters. The peaks that pass a Poisson *p* values threshold of 0.1 were used for further analyses^28^^,^^42^. In order to obtain reliable regions of enrichment, the regions detected in more than two samples were selected and aligned to define the consensus regions using DiffBind R package (v. 2.4.8)^43^. The reads in the consensus regions were counted and DESeq2 was applied to identify DMRs^44^. The DMRs were defined as *p* values of ≤.05 and only DMRs in promoter region (≤3 K from transcription start site) were selected. Then genes with the DMRs repressed by H3K9me3 in YAC and derepressed by APQ treatment were defined as seed genes for further analyses.

### Gene set analysis and construction of network model

We performed the over-representation analyses of gene ontology biological processes (GOBPs), Kyoto encyclopedia of genes and genomes (KEGG) pathways, and Reactome pathways using ConsensusPathDB^28^^,^^45^. The enriched sets of GOBPs and the pathways by the seed genes were identified as *p* values of ≤.05. Ingenuity pathway analysis (IPA; QIAGEN, Redwood City, CA) was used to construct a network model from the seed genes. The network analysis algorithm of IPA was applied to identify networks of regulatory events affected by the seed genes and their interacting genes. The importance of the genes in the network was computed as degree (*K*) and betweenness (*B*) centrality using CentiScaPe (v. 2.1), and the non-seed genes with *B* = 0 or *K* = 1 were pruned^46^. The genes in the same GOBP or pathway were grouped into the same module and labelled by the corresponding GOBP or pathway term. Cytoscape (v. 3.2.1) was used to visualise the network^28^^,^^47^.

### Statistics

Data were represented as the mean ± standard error of the mean (SEM). For single comparison, the significance of differences between means was assessed by Student's *t* test; for multiple comparisons, data were analysed by ANOVA followed by Fisher’s protected least significant difference (LSD) test using StatView 4 (Abacus Concepts, Berkeley, CA) as described previously. Data were considered significant at a value of *p*< .05.

## Results and discussion

### Identification of APQ through primary screening using Tet(Doxy)-inducible ESET cell line

**Figure 2.**
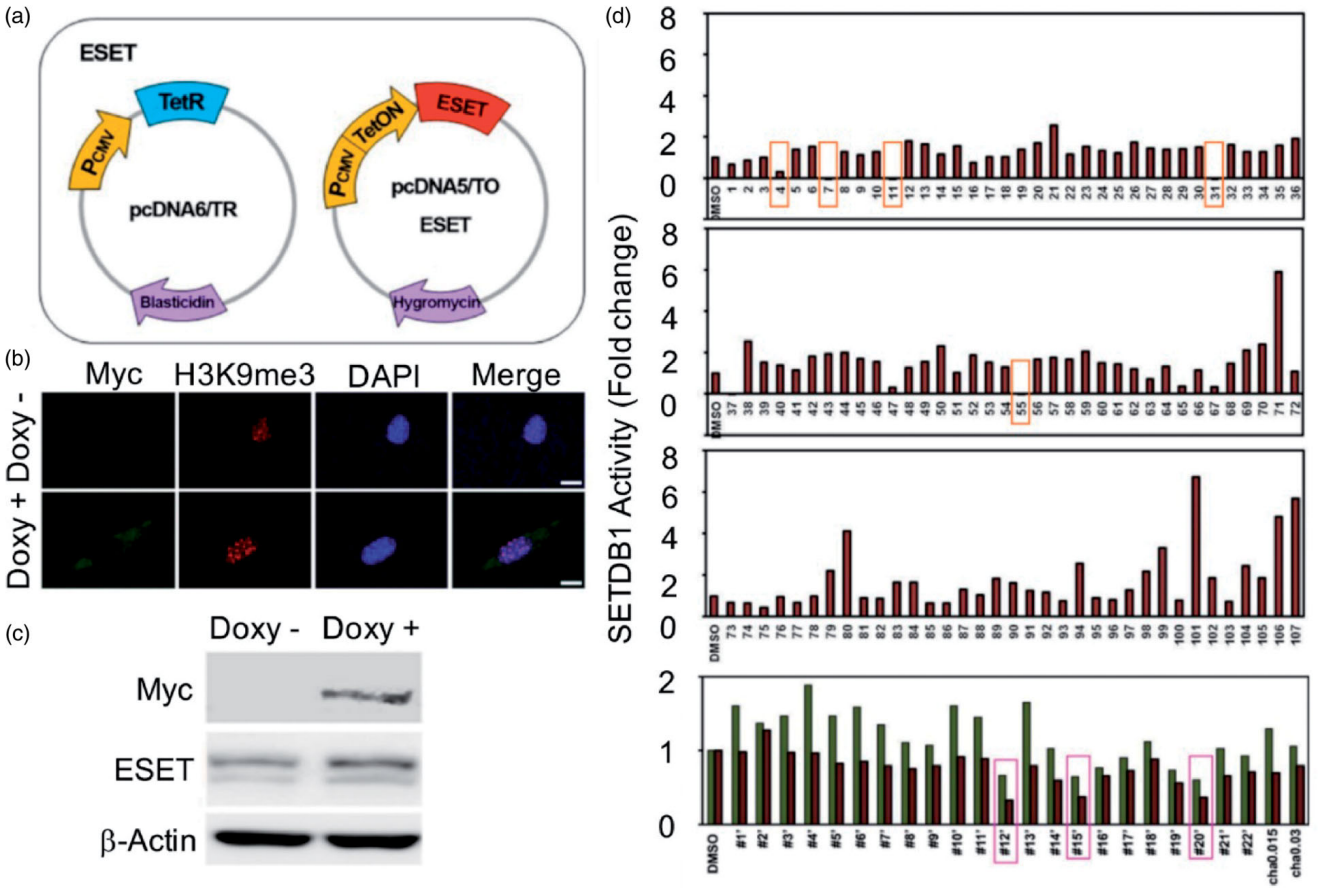
Primary screening of our compounds via cell-based and dot blot assay. (a) A diagram representing a generation of Tet-inducible SETDB1 cell line. (b, c) SETDB1 induction by doxycycline (Doxy) increased H3K9me3 level. (d) SETDB1 inhibitor candidates were determined by the dot blot assay. Inhibitors were treated to cells (in that SETDB1 was already induced by Doxy for 24 h) for 12 h. Histone protein was isolated by acidic extraction method and H3K9me3 level.

To identify candidate compounds that possess inhibitory activity against SETDB1, we have employed the previously developed dot blot assay using tetracycline (TET)-inducible SETDB1 cell line (Figure 2(a–c))^28^. When the cell line was treated with Doxy, exogenous Myc-tagged SETDB1 protein expression was induced followed by an increase of H3K9me3 level (Figure 2(b,c)). We pre-treated small compounds from the in-house chemical library and analysed the level of H3K9me3 from histone extracts. In this primary screening, several compounds having quinoline scaffold showed relatively good SETDB1 inhibitory activities (Figure 2(d)).

**Scheme 1.**
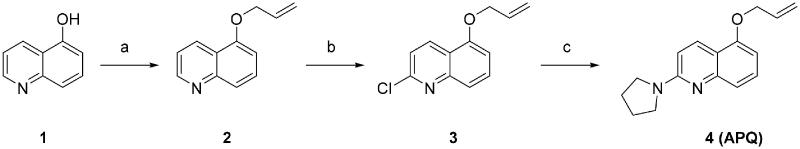
Synthesis of APQ. Reagents and conditions: (a) NaH, allyl bromide, 0–23 °C, 40 min, 92%. (b) (i) *m*CPBA, CH_2_Cl_2_, 0–23 °C, 2 h; (ii) POCl_3_, CH_2_Cl_2_, 50 °C, 7 h, 39%. (c) Pyrrolidine (neat), 90 °C, 12 h, 76%.

Among the 129 compounds tested, we have identified 5-allyloxy-2-(pyrrolidin-1-yl)quinoline (APQ) 4 as a lead compound (Scheme 1). The structure of APQ consists of three fragments including quinoline core structure, allyloxy group, and pyrrolidine. For further biological evaluation of this compound, we prepared it in a large scale following the procedures demonstrated in Scheme 1. Allylation of 5-hydroxyquinoline with allyl bromide in the presence of sodium hydride produced 5-allyloxyquinoline 2 in 92%. Treatment of 2 with mCPBA followed by POCl_3_ produced 2-chloroquinoline 3. Finally, nucleophilic aromatic substitution (S_N_Ar) of 2-chloroquinoline 3 with pyrrolidine afforded the desired compound APQ in good yield.

### Docking study

**Figure 3.**
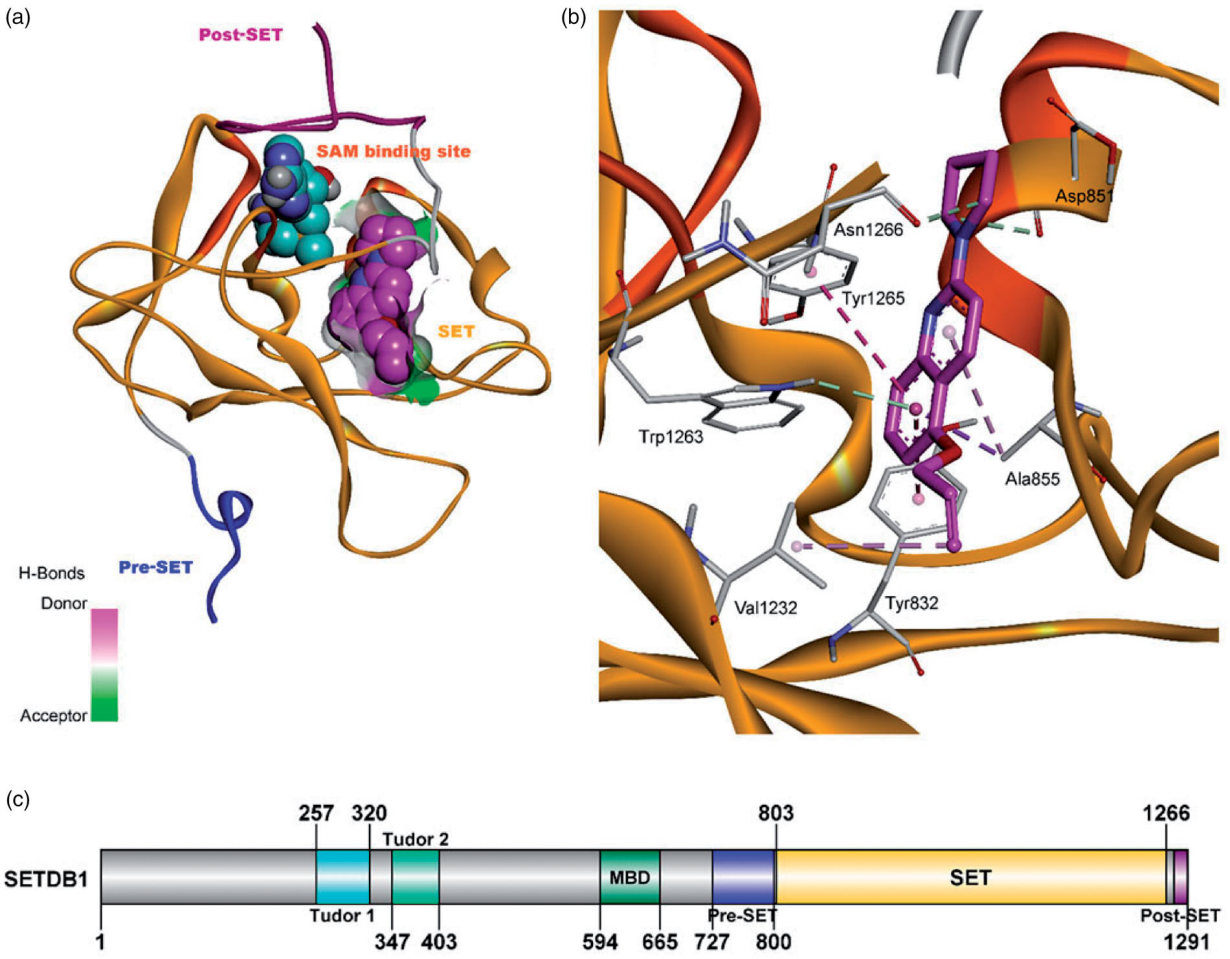
APQ ligand interaction diagram inside the SET domain. (a) SET domain represented in ribbon format. APQ and SAM showed by space-filling cpk presentation in magenta and blue colour, respectively. The hydrogen bond donor–acceptor surface area has shown around APQ. (b) APQ in magenta stick format interacts with Tyr1265, Ala855, Tyr832, Val1232, Asp851, and Asn1266. Binding site residues showed in white stick format. Pi-pi stacking, pi-sigma, pi-alkyl, and hydrophobic interactions are shown in pink, purple, light pink, and light green colour, respectively. (c) Schematic representation of SETDB1 domain organisation.

Following the discovery of APQ, we tried to obtain some insight as to how APQ could interact with the SETDB1 methyltransferase. In particular, it is crucial to understand whether APQ would fit into substrate binding domain or SAM binding domain for further optimisation of APQ. Thus, APQ molecule docked inside our homology model of SETDB1/ESET domain using the Gold suite-5.2 software^29^. Discovery studio 2018 software was used for the visualisation and image preparation. SET domain has binding sites for SAM and histone tail containing lysine residue. SAM binding pocket has connecting pore for lysine binding pocket. In the docking study, we have observed that APQ molecule binds in the connecting pore of lysine binding site and SAM binding pocket with PLP score of 70.27, as seen in Figure 3(a). To understand the exact distance of APQ binding with respect to SAM, we have taken SAM binding orientation from PDB ID 2R3A^48^ which is one of the template used in homology model preparation. Surface area continuing above the hydrophobic surface around APQ shows the connecting region of these two pockets. Figure 3(a) shows that APQ can fit inside the narrow pocket of our homology model due to its small size and planar confirmation.

APQ has shown several interactions with amino acid residues in the binding site. Quinoline ring intersects with pi-pi-T-shaped interactions with two tyrosine residues Tyr1265 and Tyr832 (Figure 3(b)). Additionally, it showed pi-sigma and week pi-alkyl contacts with Ala855. Trp1263 also has hydrophobic interactions with quinoline ring. Pyrrolidine ring interacted with Asn1266 and Asp851 by hydrophobic interactions. Alkene group has shown alkyl (hydrophobic) contacts with Val1232. Overall from the modelling study, we observed that APQ has potential to block the methylation process of lysine residue by occupying the binding site of lysine inside the catalytic domain.

### APQ reduces H3K9 trimethylation and SETDB1 expression

**Figure 4.**
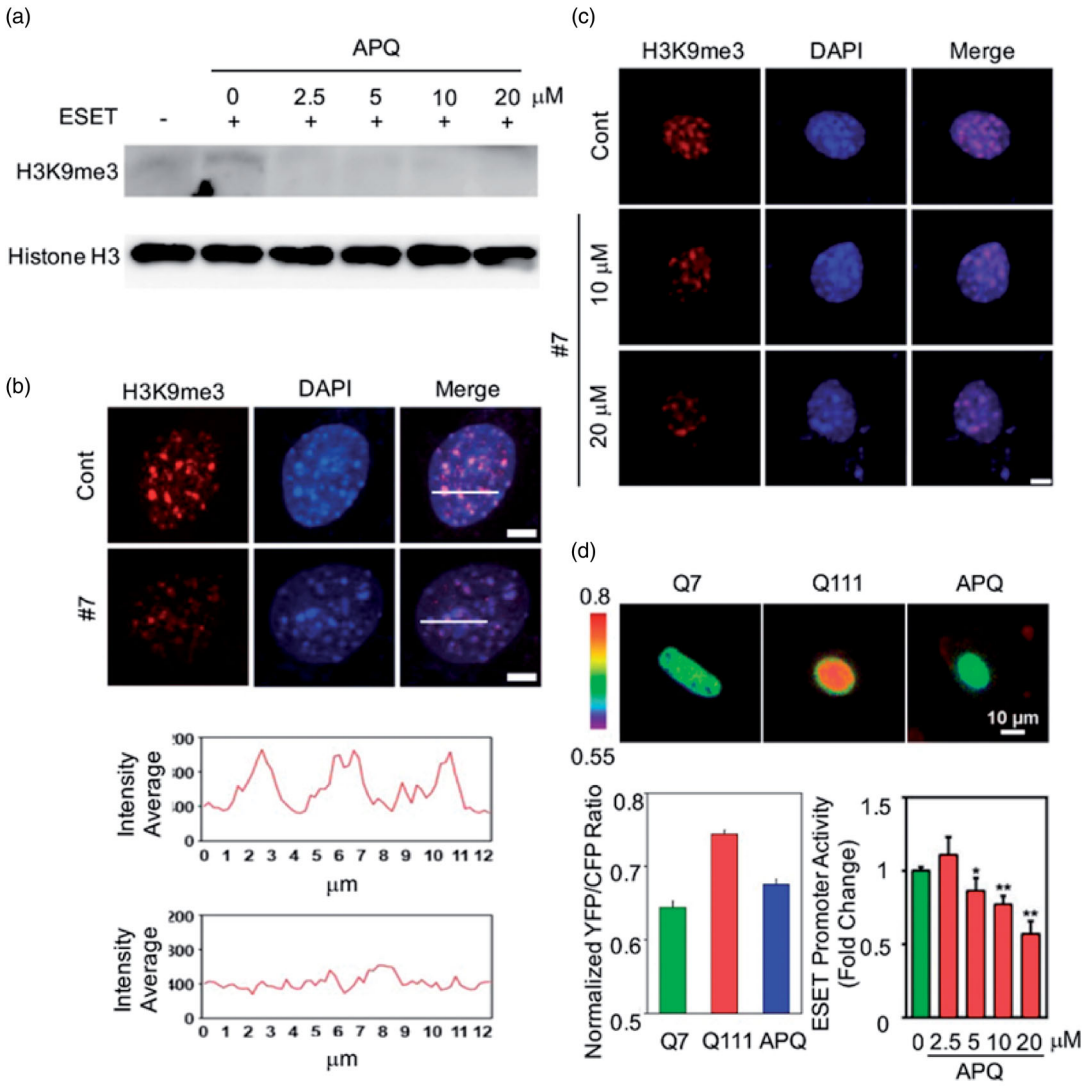
APQ reduces H3K9me3 level in HD striatal cells. (a) APQ decreases the level of H3K9me3 in HD (*Q111/Q111*) striatal cells. (b, c) APQ reduces the immunoreactivity of H3K9me3 (*Q111/Q111*) striatal cells. (d) FRET imaging analysis showing that APQ effectively modulates FRET/ECFP ratios of H3K9 methylation in Q111. APQ represses the transcriptional activity of *Setdb1/Eset* in a dose-dependent manner. The data represents the average of three separate experiments. Significantly different at *p< .05, **p< .001.

In order to further verify the effect of APQ on SETDB1 activity, the APQ dose-dependent experiments were performed and analysed H3K9me3 level by Western blot analysis (Figure 4). APQ significantly decreased SETDB1 protein levels in a dose-dependent manner in HD (Q111) cell and reduced the level of H3K9me3 level as well (Figure 4(a,b)). Confocal microscopy showed that APQ considerably decreased H3K9me3 immunoreactivity in HD cells (Figure 4(c)). When the Q111 cells were treated with APQ, we confirmed that the level of H3K9 trimethylation was significantly decreased, which is comparable to the trimethylation level of the Q7 control cells. In addition, live cell imaging analysis of the effects of APQ was performed using FRET-based H3K9 methyltransferase biosensors (Figure 4(d)). As previously described^29^, the H3K9me3 level in Q111 cells was highly increased compared to that in Q7 control cell. Moreover, the promoter assay was carried out. Thus, neuronal cells transiently transfected with SETDB1 promoter construct were treated with APQ in different concentrations from 0 to 20 µM. APQ represses the transcriptional activity of SETDB1 in a dose-dependent manner (Figure 4(e)).

### Enzyme inhibitory activity (chemiluminescence assay) and cell viability

Next, we evaluated the inhibitory activity of our compound against SETDB1 enzyme by comparing with a well-known methyltransferase inhibitor (substrate) using chemo-luminescence assay (Figure S1a). We prepared a mixture of histone H3 substrate, SAM, and diluted SETDB1 enzyme in buffer solution, which was incubated with increasing concentrations of APQ. The inhibitory activity at each concentration was recorded by detecting chemiluminescence signal after treatment of H3K9 antibody followed by addition of the HRP substrate. The SETDB1 activity was inhibited in a dose-dependent manner although the IC_50_ value of APQ is only as low as 65 µM. Additionally, the cell viability of APQ was tested by MTT assay (Figure S1b). Thus, STHdhQ7/7 (wild type) cells were cultured with varying concentrations of APQ for 24 h. The result indicated that APQ did not show any cytotoxicity at the concentration range of 1–100 µM. Therefore, APQ not only modulates the promoters to reduce the expression level of SETDB1, but also inhibits the enzymatic activity to reduce the trimethylation of H3K9.

### The pharmacokinetic properties of APQ

In order to examine the pharmacological properties SETDB1/ESET inhibitor, 10 mg/kg of APQ were intravenously and orally administered to Sprague-Dawley (SD) rats. Table 1 summarises the results. Importantly, 0.4066 µg/ml of APQ was detected in the brain while 0.10 µg/ml of APQ was detected in the plasma 2 h after the intravenous injection. It is well known that the blood–brain barrier (BBB) is a key obstacle impeding the entry of drugs into the brain. The high and sustained brain-to-plasma (B/P) ratio of APQ strongly supports its potential as an epigenetic drug for the treatment of brain disorders. In addition, APQ has a sustained plasma half-life after the intravenous and oral administration (Figure S2).

**Table 1.** Mean (±SD^a^) pharmacokinetic parameters after intravenous (*n* = 5) and oral (*n* = 4) administration of APQ (10 mg/kg) to Sprague-Dawley (SD) male rats.

Plasma	Intravenous		Oral
AUC_0–∞_ (μg min/ml)	55.38 ± 7.70		3.70 ± 0.89
AUC_last_ (μg min/ml)	51.49 ± 7.41		3.64 ± 0.86
Terminal half-life (min)	152.87 ± 6.06		94.69 ± 13.4
*C*_max_ (μg /ml)	–		0.04 ± 0.01
*T*_max_ (min)	–		60 (60–60)^b^
CL (ml/min/kg)	183.46 ± 26.07		–
MRT (min)	95.98 ± 6.84		–
*V*_ss_ (ml/kg)	25601.76 ± 5195.12		–
Ae (%)	0.00		0.00
Plasma concentration (μg/ml) at 2 h	0.10 ± 0.01		0.01 ± 0.00
Plasma concentration (μg/ml) at 8 h	0.02 ± 0.00		0.00 ± 0.00
Brain concentration (μg/ml) at 2 h	0.4066 ± 0.0813		0.0037 ± 0.0011
Brain-to-plasma (B/P) ratio at 2 h	4.18		0.44
*F* (%)		6.7	

AUC_0–∞_: total area under the plasma concentration–time curve from time zero to time infinity; AUC_last_: total area under the plasma concentration–time curve from time zero to last measured time; *C*_max_: peak plasma concentration; *T*_max_: time to reach *C*_max_; CL: time-averaged total body clearance; MRT: mean residence time; *V*_ss_: apparent volume of distribution at steady state; Ae: excreted amount; *F*: bioavailability.

^a^ SD: standard deviation.

^b^ Median (range) for *T*_max_.

### *In vivo* effects of APQ on behaviours and neuropathology in a HD mouse model

**Figure 5.**
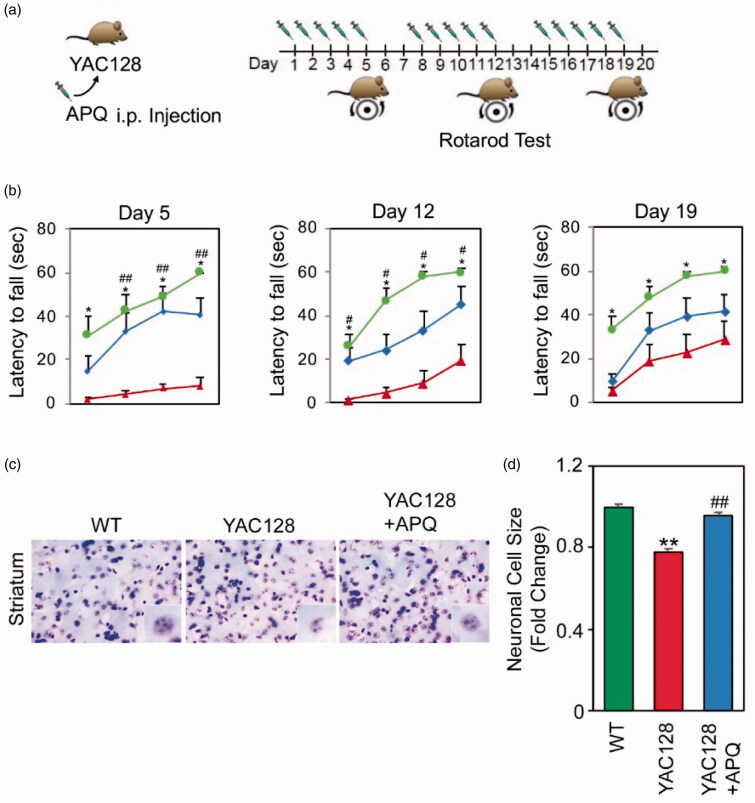
APQ improves motor behaviour and neuronal atrophy in HD transgenic (YAC128) mice. (a) A scheme showing APQ administration and rotarod test schedule. (b) Rotarod test showed that APQ administration improves motor function in YAC128 mice. Significantly different from WT at *p<.05; **p<.01. Significantly different from YAC128 mice at ^#^*p*<.05. (c) Nissl staining showed that APQ restored the size of medium spiny neurons in the striatum of YAC128 mice. (d) Quantitative analysis showed that APQ significantly increased the size of medium spiny neurons in the striatum of YAC128 mice. Significantly different from WT at **p<.01. Significantly different from YAC128 mice at ^##^*p*<.01.

To assess the effects of APQ, a novel compound targeting SETDB1, on the locomotor activity in HD mouse model, we conducted a rotarod test at 15 months of age (Figure 5(a)). Male YAC-128 transgenic mice were administered with APQ by i.p. injection five times a week at the same day for all groups. After five days, motor behaviour of YAC128 mice was significantly improved compared to vehicle treated YAC128 mice. Repeated administration of APQ continued to recover the motor performance in YAC128 mice (Figure 5(b)). The improvement of motor activity in the YAC128 mice treated with APQ for additional five days was also persisted although the motor performance was slightly increased in the vehicle-treated YAC128 mice due to learning experience induced by repeated tests. Otherwise, the Nissl staining (cresyl violet staining) of striatal neurons showed distinct neuronal atrophy in YAC128 mice compared to WT mice, which was significantly restored in APQ treated YAC128 mice (Figure 5(c)).

**Figure 6.**
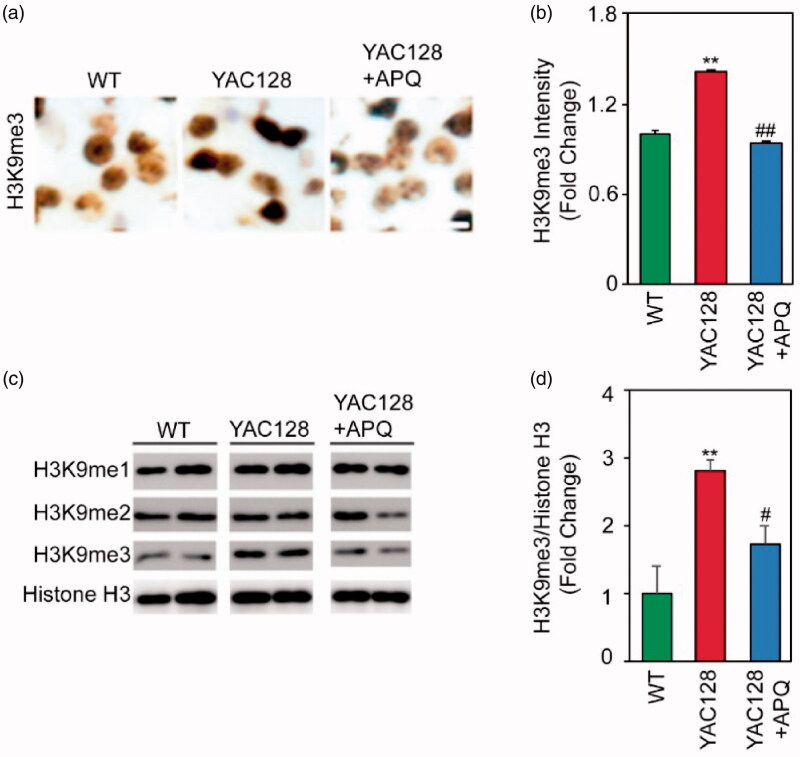
SETDB1 inhibitor (APQ) reduces H3K9me3 immunoreactivity and level in the striatum of HD transgenic (YAC128) mice. (a) APQ administration significantly decreased medium H3K9me3 immunoreactivity in the striatum of YAC128 mice (*n* = 5) compared to vehicle-treated YAC128 mice (*n* = 5). (b) APQ administration significantly reduced H3K9me3 intensity in medium spiny neurons in the striatum of YAC128 mice. Significantly different from WT at **p<.01. Significantly different from YAC128 mice at ^##^*p*<.01. (c) Acid histone extraction and Western blot analysis showed that APQ selectively decreased H3K9me3 level but not H3K9me1 and H3K9me2 levels in the striatum of YAC128 mice compared to vehicle-treated YAC128 mice. (d) Densitometry analysis showed that APQ significantly reduced H3K9me3 level in the striatum of YAC128 mice. H3K9me3 levels were normalised to histone H3 levels. Significantly different from WT at **p<.01. Significantly different from YAC128 mice at ^#^*p*<.05.

To further investigate the effects of APQ on epigenetic and neuropathological changes in HD mouse model, we performed immunohistochemistry analysis of coronal brain sections of WT, YAC128, and APQ-treated YAC 128 mice (Figure 6). While H3K9me3 immunoreactivity was increased in striatal neurons in YAC128 mice, APQ significantly reduced H3K9me3 immunoreactivity in YAC128 mice, which is comparable to the level of WT controls (Figure 6(a,b)). Consistent with these alteration, Western blot analysis verified that APQ selectively decreased H3K9me3 levels but not H3K9me1 or H3K9me2 levels in the striatum of YAC128 mice compared to vehicle-treated YAC128 mice (Figure 6(c)). Densitometry analysis presented that APQ significantly reduced H3K9me3 in YAC128 mice compared to vehicle-treated YAC128 mice (Figure 6(d)).

**Figure 7.**
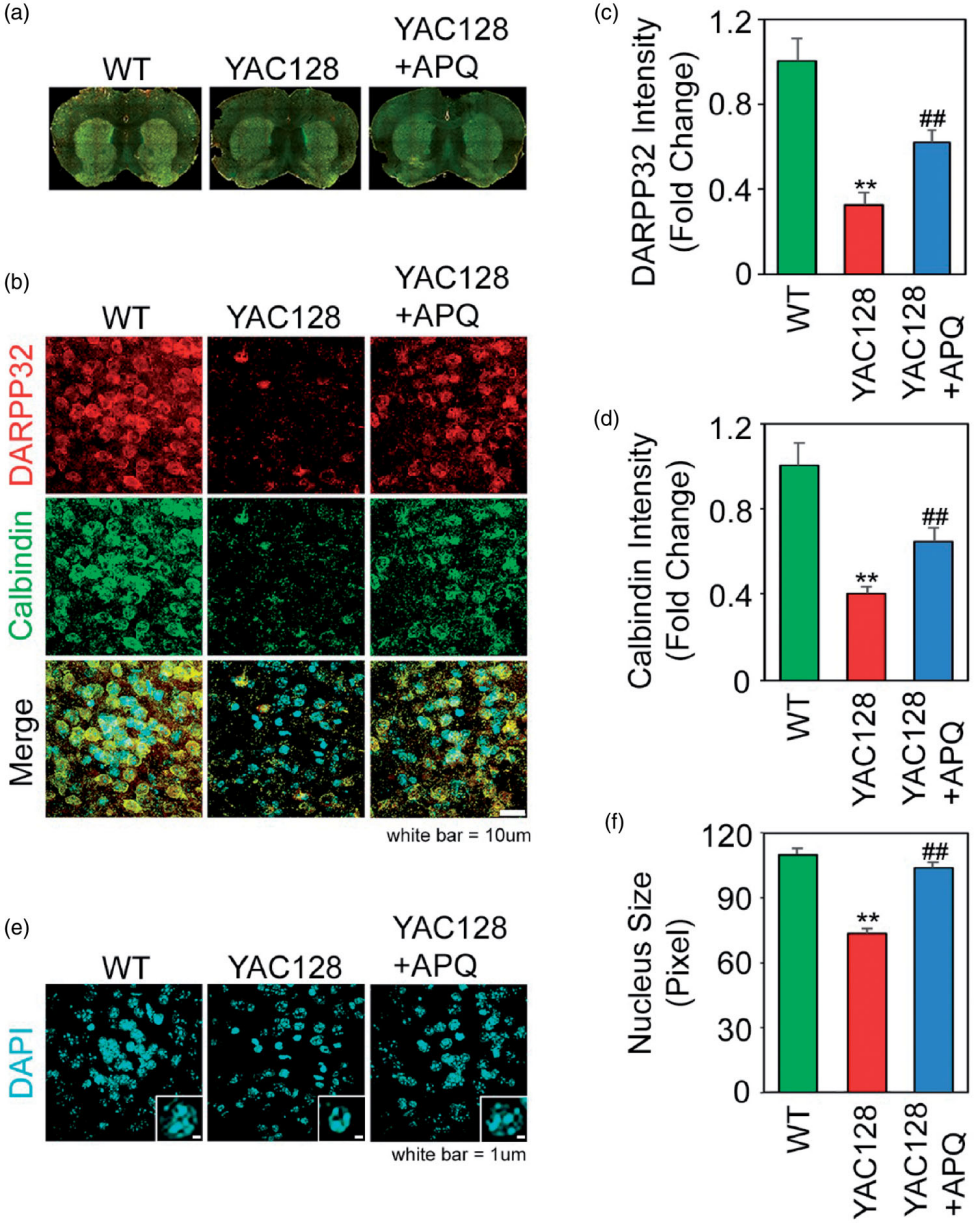
SETDB1 inhibitor (APQ) modulates medium spiny neuronal activity in the striatum of HD transgenic (YAC128) mice. (a, b) Confocal microscopy showed that APQ administration restored DARPP32 and calbindin immunoreactivity in the striatum of YAC128 mice. (c) Densitometry analysis showed that APQ significantly improved DARPP32 level in the striatum of YAC128 mice. Significantly different from WT at **p<.01. Significantly different from YAC128 mice at ^##^*p*<.01. (d) Densitometry analysis showed that APQ significantly improved calbindin level in the striatum of YAC128 mice. (e) DAPI staining showed that APQ rescued the nuclei size in the striatum of YAC128 mice. (f) Densitometry analysis showed that APQ significantly improved the nuclei size in the striatum of YAC128 mice. Significantly different from WT at **p<.01. Significantly different from YAC128 mice at ^##^*p*<.01.

DARPP-32 is a dopamine- and cAMP-regulated neuronal phosphoprotein, which plays an important role in dopamine neurotransmission. Its expression is severely impaired in the striatum of YAC128 mice compared with that of WT mice. Importantly, we observed that APQ administration considerably improved the DARPP-32 expression in in the striatum of YAC128 mice (Figure 7(a,b)). Similarly, the reduced immunoreactive intensity of calbindin stained neurons in YAC-128 mice was increased after treatment of YAC-128 mice with APQ (Figure 7(c,d)). Furthermore, analysis of DAPI-stained tissue sections of YAC128 mice indicated that APQ treatment restores the nuclei size of medium spiny neurons (Figure 7(e)).

### APQ alters the association of H3K9me3 with the promoter region of epigenomes in HD transgenic (YAC128) mice

**Figure 8.**
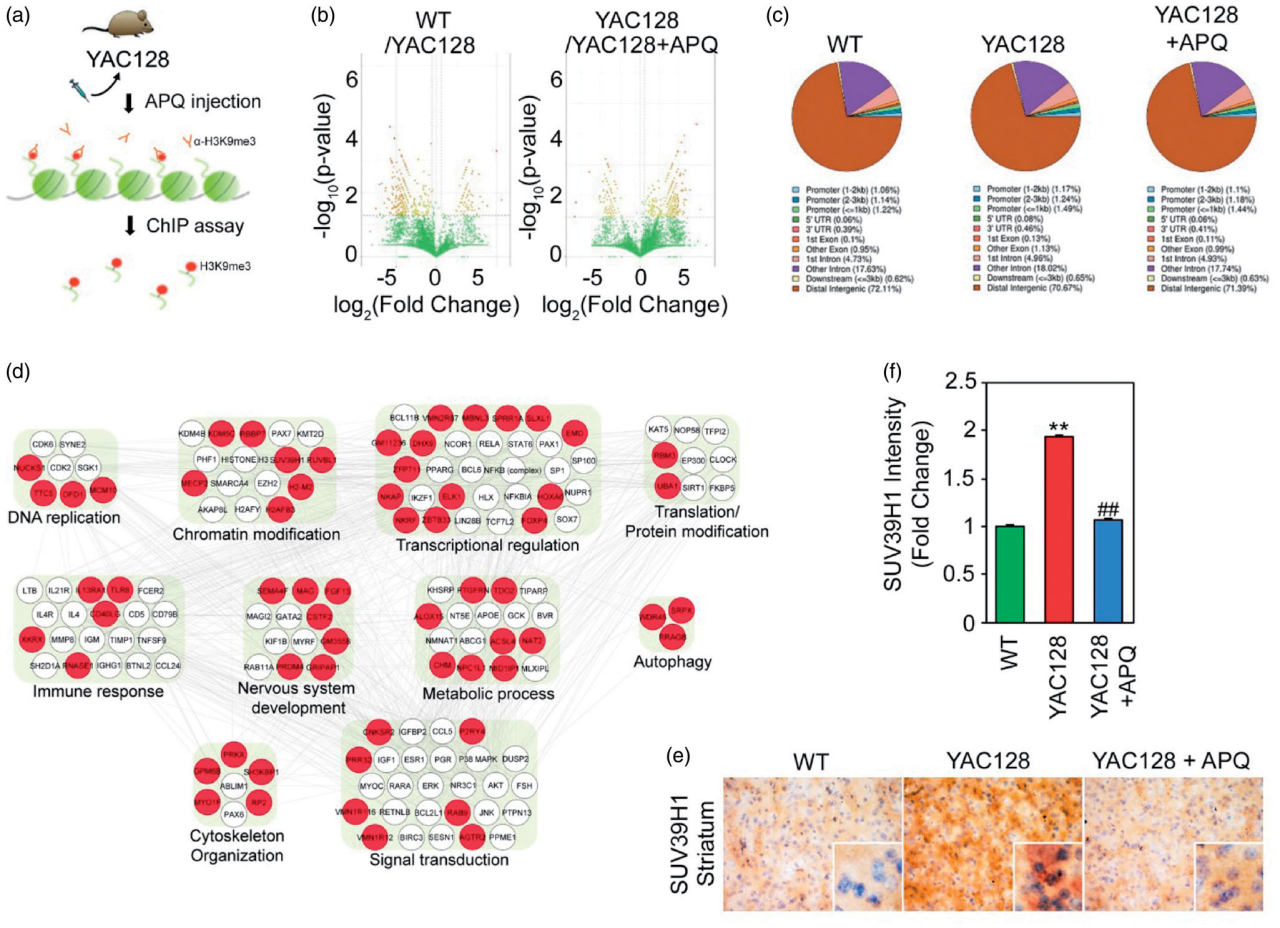
SETDB1 inhibitor (APQ) modulates H3K9me3-landscaped epigenomes in the striatum of HD transgenic (YAC128) mice. (a) A scheme illustrating H3K9me-ChIP sequencing analysis in APQ-administered YAC128 mice. (b) Volcano plots presented that H3K9me-landscaped epigenomes are altered in APQ-administered YAC128 mice. (c) Pie graphs exhibited that APQ affects the landscaping of H3K9me in the promoter regions of epigenomes in YAC128 mice. (d) Network analysis showed that APQ modulates remodelling of H3K9me-landscaped epigenomes (red nodules) that are associated with chromatin modification, transcription regulation, and nervous system development pathway in YAC128 mice. (e) Immunohistochemistry showed that APQ reduces SUV39H1 immunoreactivity (brown) in the striatal neurons of YAC128 mice. The nuclei (blue) were counterstained with haematoxylin. (f) Densitometry analysis showed that APQ significantly reduced SUV39H1 level in the striatal neurons of YAC128 mice. Significantly different from WT at **p<.01. Significantly different from YAC128 mice at ^##^*p*<.01.

In order to determine how the occupancy of H3K9me3 is changed in the promoter of genes and whether APQ modulates H3K9me3-enriched genes in the medium spiny neurons of YAC128 mice, we performed H3K9me3-ChIP sequencing followed by GO and biological network analysis (Figure 8(a), Table S1). Among the read peaks, a total of H3K9me3-enriched 71735 sites were commonly detected using MACS in WT, vehicle-treated YAC128, and APQ-treated YAC128 mice (using DiffBind R package). The reads were re-counted in the 71,735 sites (using DESeq2) and the H3K9me3-enriched sites in promoter region (≤3 K), with *p* values ≤.01 were selected. Then the H3K9me3-enriched sites with 1.5-fold higher in vehicle-treated YAC128 compared to WT and 1.5-fold lower in vehicle-treated YAC128 compared to APQ-treated YAC128 were finally selected. APQ treatment led to a 1.5-fold decrease of H3K9me3 occupancy in a total of 7060 targets in YAC mice (Figure 8(b)). Pie graph analysis showed that the H3K9me3 occupancy in the promoter region (–1 k to –3 kb) is reduced in APQ-treated YAC128 mice compared to vehicle-treated YAC128 mice (Figure 8(c)).

### Network analysis shows that APQ modulates epigenomes associated with chromatin modifications and transcription regulation

To define the H3K9me3-landscaped epigenome and its modulation by APQ, we ran a functional enrichment analysis of GOBPs and presented a biological network illustrating epigenome interactions among the most representative GOBPs. GOBP analysis showed that these epigenome targets are associated with sensory protection and neurological system processes (Figure 8(d)). The network analysis showed that APQ alters the H3K9me3 occupancy in the promoter region of a total of 68 epigenome in YAC128 mice. Red nodes indicate the genes that were repressed by H3K9me3 in YAC128 mice and derepressed by APQ treatment in their promoter region (Figure 8(d)). White nodes are interacting genes with the seed genes identified by the network analysis algorithm of IPA. The network represented dense connections between the epigenome associated with chromatin modification (such as *Suv39h1*, *Mecp2*, *KDM5c*, etc.), transcription regulation (such as *Elk1*, *Foxp4*, *Hoxa6*, *Dhx9*, etc.), metabolic process (such as *Acsl4*, *Fbp2*, *Nat2*, etc.), and signal transduction pathways (such as *Cnksr2*, *Dusp9*, *Rab9*, etc.) (Figure 8(d)). Especially, SUV39H1, a histone H3K9 methyltransferase, was closely associated with signalling pathways and other modules (Figure 8(d)). We further verified that SUV39H1 immunoreactivity was markedly elevated in YAC128 mice (Figure 8(e)). As we expected, APQ-administration significantly reduced the level of SUV39H1 in YAC128 mice as similar to the level in WT mice (Figure 8(f)).

## Conclusions

We identified a novel small molecule SETDB1 inhibitor and validated its neuroprotective effect on *in vitro* HD cell (*HTdh^Q111/Q11^1*) line and *in vivo* HD transgenic (YAC128) mouse model. Primary screening of a chemical library using the Dot-blot assay rapidly provided APQ as a lead compound. The homology modelling study showed that APQ is predicted to block the methylation process of lysine residue by occupying the binding site of lysine inside the catalytic domain. We observed that APQ down-regulated SETDB1 expression and reduced H3K9 trimethylation. APQ reduced both transcriptional and enzymatic activity of SETDB1 without showing any significant cytotoxicity. Most importantly, APQ restored motor behaviour in the HD mouse model through histone modification by regulation of SETDB1. It is interesting that APQ alters H3K9me3-landscaping in multiple genes that are associated with histone modification and transcription regulation processes that may be involved in the pathogenesis in HD. We have reported that a vicious cycle of H3K9me-dependent heterochromatin condensation by SETDB1 is linked to transcriptional dysfunction, neuropathological processes, and motor behavioural dysfunction in HD animal models. Importantly, the increase of H3K9me3 level directly contributes to the condensation of pericentromeric heterochromatin and it is reversibly regulated by small molecules^24^^,^^28^^,^^29^. In the current study, we verified that APQ is a *bona fide* epigenetic drug, improving motor function in the HD mouse model through the recovery of neuronal activity by reducing SETDB1 activity and H3K9me3-dependent heterochromatin plasticity. Our results show that epigenetic SETDB1 inhibition would be a potential therapeutic target for HD and provide a starting point for lead optimisation into further development of SETDB1 inhibitors for clinical trials.

## Supplementary Material

Supplemental Material
